# OA20 Differentiating Lupus Nephritis from Pre-eclampsia-A Diagnostic Dilemma

**DOI:** 10.1093/rap/rkae117.020

**Published:** 2024-11-05

**Authors:** Kapil Garg, Anupama Nandagudi

**Affiliations:** Basildon University Hospital, Mid and South Essex NHS Foundation Trust, Basildon, United Kingdom; Basildon University Hospital, Mid and South Essex NHS Foundation Trust, Basildon, United Kingdom

## Abstract

**Introduction:**

Estimated prevalence of SLE is 97/100 000 in UK adult population. Upto 60% of SLE patients develop lupus nephritis (LN). Management of LN in women of child-bearing age is particularly complex. These patients should be risk-stratified as quiescent, active and with severe end-organ damage. Pregnancy should be discouraged in severe end-organ damage. Active LN should be managed by delaying pregnancy until disease-activity is controlled. Quiescent disease patients should have optimal plan of care in place during pregnancy. Differentiation between LN flare and pre-eclampsia is also challenging in these patients due to clinical overlap especially later in pregnancy.

**Case description:**

We present a case of 33-year-old female who was diagnosed with SLE in 2011 and subsequently LN WHO class III on renal biopsy in 2013. She was treated with Rituximab followed by Azathioprine (AZA). She had developed CKD G1A1 with haematuria and proteinuria being managed under combined care of rheumatology and nephrology. She had raised anti-dsDNA at 67.3IU/ml and normal complements. She has background urine PCR 38.9 mg/mmol. Her disease was well controlled on HCQS 400 mg/day, Azathioprine 75 mg OD and Irbesartan 450 mg/day.

She had pre-pregnancy counselling and advice given on risk of LN flare and pre-eclampsia. She became pregnant in 2020 and irbesartan was switched to labetalol to manage underlying hypertension. Unfortunately, she developed high BP during 3^rd^ trimester which was managed by optimising anti-hypertensives. She developed increasing proteinuria with urine PCR 187 mg/mmol/L with normal creatinine. Her complements remained normal throughout, and anti-dsDNA titres remained stable between 50-60IU/ml. In the absence of no other SLE features, SLEDAI of 6 (proteinuria and raised dsDNA), absence of active urinary sediments and normal complements, she was treated as pre-eclampsia rather than LN. She did develop slight transaminitis (ALT 136), which normalised after stopping azathioprine. She delivered healthy baby at 38 weeks post-induction. MMF was started post-pregnancy 1.5 g/day in place of azathioprine.

Patient had second pregnancy in 2023 after pre-pregnancy counselling and explaining about high risk of pre-eclampsia. Her MMF was switched to tacrolimus 2.5 mg/day with close drug monitoring. Her ARB was changed to labetalol and HCQS was continued. She was closely monitored throughout pregnancy with stable anti-dsDNA (37 IU/ml), normal complements and low SLEDAI score. Her urine ACR was 1.8 mg/mmol/L and PCR was 69 mg/mmol/L and remained stable. BP was well controlled. Patient delivered a healthy baby at 37 weeks by induction. Post delivery at 6-weeks, tacrolimus was switched back to MMF.

**Discussion:**

Our patient had pre-eclampsia during her first pregnancy which was difficult to differentiate from LN flare. She was managed during second pregnancy with tacrolimus aiming for better disease control and control of BP. Having a controlled disease reduces risk of LN flare, risk of pre-eclampsia and confusion with other pregnancy related complications if they happen. Patient was closely followed by rheumatology, renal and obstetric teams in both her pregnancies and we had MDT discussions various times in regard to her care. Fortunately, our patient delivered healthy baby both times with no significant complications or long-term organ impairment.

New/worsening proteinuria and hypertension early in pregnancy especially with known LN will almost always represent a flare of LN. Low or low-normal complements, high anti ds-DNA titres and active urinary sediments are highly suggestive of LN flare. LN flare can happen anytime during pregnancy and hypertension might be absent. Renal biopsy is less warranted in these situations but might be considered if proteinuria is significant and likely to change management.

Pre-eclampsia occurs usually after 20 weeks of gestation with presence of hypertension, raised uric acid levels (>4.9 mg/dL) and low urinary calcium excretion (<195 mg/day). There is absence of active urinary sediments and complements would be normal. Pregnancy increases risk of LN flare and LN increases risk of pre-eclampsia. Hence both the conditions might exist concurrently.

A recent meta-analysis stated the following complications associated with pregnancy in patients with LN over the last 2 decades; gestational hypertension (odds ratio [OR], 5.65; 95% confidence interval [CI], 2.94-10.84), pre-eclampsia (OR, 2.84; 95% CI, 1.87-4.30), SLE flare (OR, 2.66; 95% CI, 1.51-4.70), LN flare (OR, 15.18; 95% CI, 5.89-39.14), and proteinuria (OR, 8.86; 95% CI 4.75-16.52).

**Key learning points:**

• Managing patients’ expectations about conception and SLE disease activity can prove challenging.

• Preconception counselling is imperative for female patients of child-bearing age with stress on risks and complications of pregnancy, medications and lupus. Risk stratification LN patients prior to planning pregnancy helps improve outcomes for mother and foetus.

• Differentiating LN flare and pre-eclampsia poses clinical challenges as management depends on correct diagnosis. Misdiagnosing LN flare in pre-eclampsia patient may expose mother and foetus to unnecessary immunosuppressants with significant side-effects. Management of pre-eclampsia is symptomatic with early delivery. Alternatively, not diagnosing LN flare in time may lead to adverse pregnancy outcomes and long-term organ damage.

• There are no biomarkers available in clinical practice currently. Reduced levels of placental growth factor later in pregnancy have predicted onset of pre-eclampsia in one study. This would need further clinical validation.

• Management of LN patients rest on multi-disciplinary approach with rheumatology, nephrology and obstetric teams’ involvement with close monitoring of disease activity and foetal well-being.

• A shared decision making with patient stressing on risk-benefit is paramount to reduce anxiety and part of good medical practice.

