# Correction to: OA20 Differentiating Lupus Nephritis from Pre-eclampsia-A Diagnostic Dilemma

**DOI:** 10.1093/rap/rkae156

**Published:** 2025-01-03

This is a correction to: Kapil Garg, Anupama Nandagudi, OA20 Differentiating Lupus Nephritis from Pre-eclampsia-A Diagnostic Dilemma, *Rheumatology Advances in Practice*, Volume 8, Issue Supplement_1, November 2024, rkae117.020, https://doi.org/10.1093/rap/rkae117.020

In the version originally published, the second author was incorrectly listed as Anupama Nanadagudi. This has been corrected online.

